# Heterologous Production in the *Synechocystis* Chassis Suggests the Biosynthetic Pathway of Astaxanthin in Cyanobacteria

**DOI:** 10.3390/antiox12101826

**Published:** 2023-10-03

**Authors:** Hanyu Liang, Hongjuan Chen, Xinya Liu, Zihan Wang, Pengfu Li, Shan Lu

**Affiliations:** 1 State Key Laboratory of Pharmaceutical Biotechnology, School of Life Sciences, Nanjing University, Nanjing 210023, China; 2 Shenzhen Research Institute of Nanjing University, Shenzhen 518000, China

**Keywords:** astaxanthin, β-carotene ketolase, carotene β-hydroxylase, biosynthesis, cyanobacteria, *Synechocystis* sp. PCC6803

## Abstract

Astaxanthin is a carotenoid species with the highest antioxidant capability. Its natural resource is very rare. The biosynthesis of astaxanthin from β-carotene includes a hydroxylation step and a ketolation step, for which the corresponding enzymes have been characterized in a few species. However, the sequence of these two reactions is unclear, and may vary with different organisms. In this study, we aimed to elucidate this sequence in *Synechocystis*, which is an ideal cyanobacterial synthetic biology chassis. We first silenced the endogenous carotene oxygenase gene *SyneCrtO* to avoid its possible interference in the carotenoid metabolic network. We then introduced the β-carotene ketolase gene from *Haematococcus pluvialis* (*HpBKT*) and the CrtZ-type carotene β-hydroxylase gene from *Pantoea agglomerans* (*PaCrtZ*) to this *δCrtO* strain. Our pigment analysis demonstrated that both the endogenous CrtR-type carotene hydroxylase SyneCrtR and HpBKT have the preference to use β-carotene as their substrate for hydroxylation and ketolation reactions to produce zeaxanthin and canthaxanthin, respectively. However, the endogenous SyneCrtR is not able to further catalyze the 3,3′-hydroxylation of canthaxanthin to generate astaxanthin. From our results, a higher accumulation of canthaxanthin and a much lower level of astaxanthin, as confirmed using liquid chromatography–tandem mass spectrometry analysis, were detected in our transgenic *BKT*^+^/*CrtZ*^+^/*δCrtO* cells. Therefore, we proposed that the bottleneck for the heterologous production of astaxanthin in *Synechocystis* might exist at the hydroxylation step, which requires a comprehensive screening or genetic engineering for the corresponding carotene hydroxylase to enable the industrial production of astaxanthin.

## 1. Introduction

Carotenoids (including vitamin A and pro-vitamin As), tocopherols (vitamin Es), polyphenols, and vitamin C are major plant antioxidants [1]. They have pivotal functions in plant acclimation to biotic and abiotic stresses, and are also essential phytonutrients to human beings [1]. Among these antioxidants, carotenoids are widely distributed in all photosynthetic organisms (ranging from prokaryotic cyanobacteria to higher plants) and also some non-photosynthetic bacteria and fungi [2]. In photosynthetic organisms, carotenoids are the main antioxidants in the chloroplast to scavenge the toxic reactive oxygen species (ROS) generated by photosynthesis [2]. A variety of carotenoids, such as lycopene, β-carotene, lutein, and astaxanthin, are critical for human beings, largely because of their antioxidant activities [3,4]. For example, carotenoids are known to reduce the risk of a variety of age-related diseases, such as neurological disorders and macular degeneration [5]. Because our bodies are not able to de novo synthesize carotenoids, human beings have to acquire them from mostly vegetable diets [6]. Besides their antioxidative capabilities, carotenoids also serve as major pigments for the colors of plant leaves, flowers, and fruits, and impart animal feathers, skin, flesh, and carapace with distinct colors [7].

**Figure 1:**
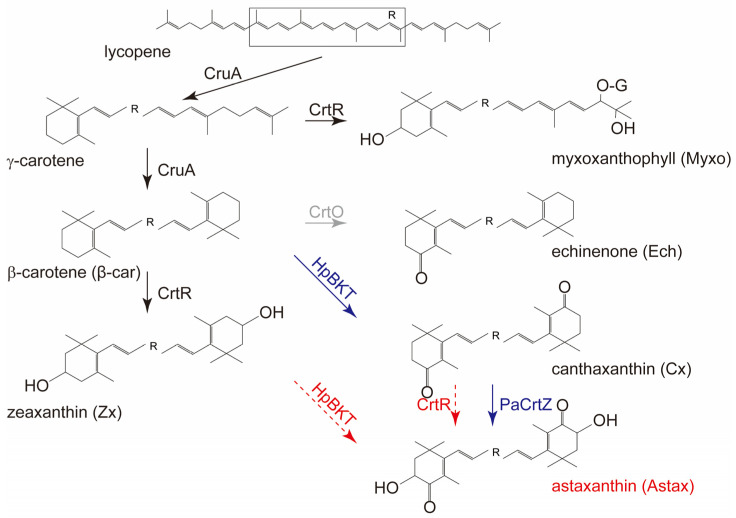
The carotenoid metabolic pathway in *Synechocystis*. The black arrows indicate the reactions catalyzed by endogenous enzymes. The gray arrow indicates the silenced conversion from β-carotene to echinenone (Ech) in our transgenic strains. Blue arrows and red dashed arrows show the reactions where we confirmed their function or not, respectively, in this study. The enzymes are endogenous lycopene β-cyclase (CruA), carotene hydroxylase (CrtR), and carotene oxygenase (CrtO) from *Synechocystis*, the transgenic β-carotene ketolase is from *Haematococcus pluvialis* (HpBKT), and the β-carotene hydroxylase is from *Pantoea agglomerans* (PaCrtZ).

Carotenoids are lipid-soluble terpenoid pigments. In higher plants, lycopene with a linear structure is the first carotenoid species with color (pink), and also the first branching point in the biosynthetic pathway (Figure 1) [8]. From lycopene, two cyclases and two hydroxylases direct the metabolic flux into two separate branches. In the β,β-branch, both ends of lycopene are β-cyclized to form β-carotene, which is then β-hydroxylated on both ends to generate zeaxanthin and further oxygenated into antheraxanthin and violaxanthin by zeaxanthin epoxidase (ZEP) (Figure 1) [9]. In the β,ε-branch, the two ends of lycopene are β- and ε-cyclized, individually, into α-carotene, which is then β- and ε-hydroxylated, respectively, to form lutein. Although carotenoids are widely distributed in nature, and Viridiplantae organisms generally share a common carotenoid profile, only zeaxanthin and β-carotene are synthesized in all photosynthetic organisms. Cyanobacteria and some red algae are not able to synthesize lutein and/or any carotenoid species of the β,β-branch beyond zeaxanthin [10,11]. Moreover, some special carotenoids are only synthesized in very limited species. For example, capsanthin and capsorubin are synthesized mainly in chili pepper (*Capsicum annuum*) [12], and astaxanthin in *Adonis aestivalis*, a Ranunculaceae herb which accumulates astaxanthin in its flowers, and the green alga *Haematococcus pluvialis* (also named as *H. lacustris*) that mainly accumulates astaxanthin in its resting form (cyst) [13,14,15].

Astaxanthin is an oxygenated carotenoid with promising antioxidant activity, which is estimated to be 100–500 times stronger than α-tocopherol and 5–15 times stronger than other carotenoids such as β-carotene [16]. Astaxanthin has been used as a neuroprotective, cardioprotective, and antitumoral chemical for the treatment of a series of diseases, such as Alzheimer’s, Parkinson’s, cardiovascular diseases, and cancer [17,18,19]. Although astaxanthin can be chemically synthesized, and the synthetic product dominates the commercial market, the impurity (e.g., different stereoisomers) largely prevents the usage of astaxanthin in the cosmetic and pharmaceutical industries [20,21]. Deciphering the metabolic pathway should shed light on the heterologous production of astaxanthin through synthetic biological approaches.

Astaxanthin has two β-rings in its molecular structure, and there is no doubt that β-carotene is a precursor for its biosynthesis [22]. Structurally, there are two modifications in astaxanthin, i.e., the 3,3′-hydroxyl groups and the 4,4′-keto groups, compared with β-carotene (Figure 1). The first attempt at elucidating its biosynthetic pathway in higher plants was achieved in *Adonis aestivalis* [14]. Two enzymes, a carotenoid β-ring 4-dehydroxygenase (CBFD) and a 4-hydroxy-β-ring 4-dehydroxygenase (HBFD), were identified to take part in the three-step conversion from β-carotene to astaxanthin, which was proposed to be catalyzed sequentially by CBFD, HBFD, and CBFD again [14]. In the marine bacterium *Paracoccus*, carotenoid oxygenase CrtW and hydroxylase CrtZ were proposed to catalyze the ketolation and hydroxylation steps between β-carotene and astaxanthin, respectively, in an unclear sequence [23,24]. In the green alga *Haematococcus pluvialis*, a β-carotene-4-dehydroxygenase, which is also named as β-carotene ketolase (HpBKT), was earlier identified to catalyze the ketolation reaction from β-carotene to canthaxanthin [24,25]. However, it is largely unknown what the preferential intermediates are for the two types of enzymes in the conversion from β-carotene to astaxanthin, i.e., β-carotene could either be firstly ketolated into echinenone and canthaxanthin, and then be hydroxylated into astaxanthin, or, in another sequence, be hydroxylated into β-cryptoxanthin and zeaxanthin first, and then be ketolated into astaxanthin (Figure 1) [26]. Recently, the cyanobacterium *Synechocystis* has been developed as a platform for identifying genes and enzymes involved in carotenoid metabolism [27]. Similarly, the cyanobacterial chassis was also used for heterologously producing astaxanthin to a level up to 29.6 mg g^−1^ dry weight (DW) astaxanthin by introducing both hydroxylase and ketolase together [28,29]. However, the sequence of the two reactions, i.e., the ketolation and the hydroxylation, is still unknown.

In cyanobacteria, there are endogenous carotenoid oxygenase (CrtO) and the CrtR-type carotene hydroxylase [27,30], both of which have the possibility of becoming involved in the biosynthesis of astaxanthin together with exogenous transgenes. For example, the *CrtO* gene from *Haematococcus pluvialis* was found to facilitate the biosynthesis of astaxanthin in tobacco flowers, suggesting that the transgenic HpCrtO, not the endogenous cytochrome P450-type nor the non-heme carotene hydroxylase, is able to carry out the ketolation reaction [20].

In this study, we tried to elucidate the reaction sequence of the last two metabolic steps for astaxanthin biosynthesis using *Synechocystis* by silencing the endogenous CrtO gene (*SyneCrtO*) and introducing *HpBKT* and the CrtZ-type carotene β-hydroxylase gene from *Pantoea agglomerans* (*PaCrtZ*), of which the 3-hydroxylation activity on β-carotene was recently reported [31]. Compared with green algae and land plants, cyanobacteria have a very simple carotenoid biosynthetic pathway, which produces only a few carotenoid constituents. This makes it quite easy to detect and analyze the functions of transgenes without the interference and/or redundancy from endogenous genes. Benefiting from the advantages of being able to grow both auto- and heterotrophically, cyanobacterium does not need an organic carbon source, which is required for the fermentation of bacterial and yeast cells, but is also able to grow much faster than green algae and land plants. This makes cyanobacteria an ideal synthetic biology chassis [32]. An elucidation of the reaction sequence would help to identify the potential bottleneck in the heterologous synthesis of astaxanthin, and also contribute to its industrial production as a major supply of this antioxidant.

## 2. Materials and Methods

### 2.1. Strains and Growth Conditions

The cyanobacterial *Synechocystis* sp. PCC6803 strain was originally obtained from the Pasteur Culture Collection (Paris, France), and then subcultured in our laboratory at Nanjing University (Nanjing, China) on BG11 plates or in liquid BG11 medium under continuous illumination with a light intensity of 30 μmol photons m^−2^ s^−1^ at 28 °C [27,33]. Cell growth in the liquid culture was monitored at OD_750_ using a spectrophotometer (Multiskan Go, Thermo Scientific, Waltham, MA, USA).

### 2.2. Gene Cloning and Vector Construction

The *Escherichia coli* strain TOP10 (TaKaRa, Shiga, Japan) was used as a host strain for gene cloning. For PCR amplification, the high-fidelity PrimeSTAR HS DNA polymerase (TaKaRa) was used throughout the experiments. All primers used in this study are listed in Supplemental Table S1.

**Figure 2:**
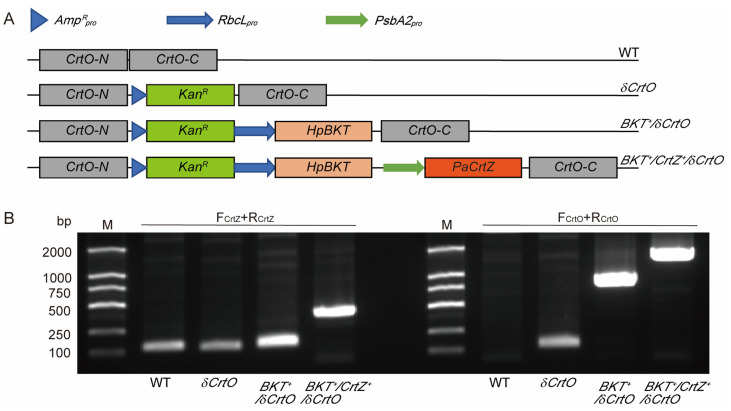
Vector construction and verification. (**A**) Designs of the constructs. The wild-type (WT) *CrtO* gene structure in *Synechocystis* genome. CrtO-N and CrtO-C indicate the 5′- and 3′-ends encoding the N- and C-termini of CrtO, respectively, which are the target regions for homologous recombination. A kanamycin resistance gene (*Kan^R^*) driven by the promoter of an ampicillin resistance gene (*Amp^R^_pro_*) was used as a selection marker in this study to generate the *δCrtO* strain, from which the β-carotene ketolase gene from *Haematococcus pluvialis* (*HpBKT*) driven by the promoter of Rubisco large subunit gene (*RbcL_pro_*) was further incorporated to generate the *BKT^+^/δCrtO* strain. A β-carotene hydroxylase gene from *Pantoea agglomerans* (*PaCrtZ*) after the D1 protein gene promoter (*PsbA2_pro_*) was further introduced to generate the *BKT^+^/CrtZ^+^/δCrtO* strain. (**B**) PCR amplification confirms the aforementioned constructs. Primers are the forward (F_CrtZ_) and reverse (R_CrtZ_) ones for *PaCrtZ*, and the forward (F_CrtO_) and reverse (R_CrtO_) ones in the CrtO-N and CrtO-C regions in (**A**). Genomic DNA was extracted from the transgenic cyanobacterial cells growing on BG11 plates supplemented with kanamycin, and then used for PCR amplification using the indicated primer pairs. DL2000 DNA marker (M) was used to indicate fragment sizes.

The design of the constructs is illustrated in Figure 2A. In brief, sequences of *HpBKT* (GenBank accession No. BAA08300.1) and *PaCrtZ* (GenBank accession No. AAA64983.1) were downloaded from GenBank and directly synthesized by Convenience Biology (Changzhou, China). The promoters of the genes for *Synechocystis* Rubisco large subunit (*RbcL_pro_*) and the D1 protein of photosystem II (*PsbA2_pro_*) were amplified from a cDNA pool prepared from *Synechocystis* using the PrimeScript 1st strand cDNA synthesis kit (TaKaRa) [34,35]. The kanamycin resistance gene *Kan^R^* driven by an ampicillin resistance gene promoter (*Amp^R^_pro_*) was used as a selection marker, as described previously [27]. Different genes or gene combinations were assembled into pUC19 using a ClonExpress MultiS One-Step Cloning Kit (Vazyme, Nanjing, China), according to our previous report [27]. Homologous fragments for transforming *Synechocystis* through recombination were designed to target (and thus interrupt) the endogenous *SyneCrtO* gene [27]. All constructs were sequenced to confirm their correctness.

### 2.3. Synechocystis Transformation and Mutant Screening

For *Synechocystis* transformation, the liquid culture at the logarithmic growth stage (OD_750_ = 0.5–0.8) was used. In brief, 10 mL cells were collected using centrifugation at 1107× *g* for 5 min and resuspended in 5 mL BG11 medium. One mL of suspended cells was then mixed with 3–5 μg each of the plasmids, followed by an overnight incubation at 28 °C in the dark. The incubated cells were then plated on a non-selective BG11 plate for about 2 d under constant light. The plates were then lifted with a sterilized spatula and 1 mL of kanamycin at 15 μg mL^−1^ was applied to the bottom of the Petri dishes. Plates were sealed to avoid drying out and were further incubated under continuous illumination at a light intensity of 30 μmol photons m^−2^ s^−1^ at 28 °C [36]. Colonies that appeared after 2 to 3 weeks were picked, further confirmed using PCR, and used for subsequent analysis. Positive transformants were maintained on BG11 agar plates containing 20 μg mL^−1^ kanamycin [36].

### 2.4. Carotenoid Extraction and Analysis

Two mL of the *Synechocystis* liquid culture at OD_750_ = 1.0 was centrifuged at 8000× *g* for 2 min. Pelleted cells were extracted using 400 μL of a mixture of chloroform and methanol (2:1, *v*/*v*). After centrifugation at 12,000× *g* for 10 min, the extract was dried under a nitrogen stream. The concentrated sample was re-dissolved in 200 μL ethyl acetate and used for further analysis [37].

An Agilent 1260 high-performance liquid chromatography (HPLC) system (Agilent, Santa Clara, CA, USA) equipped with a diode-array detector was used for pigment analysis. Pigments were separated on a Spherisorb ODS2 column (4.6 × 250 mm, 5 μm, Waters, Milford, MA, USA) at 30 °C. Samples were eluted with a 45 min linear gradient from 100% mobile phase A (acetonitrile: water: triethylamine = 9:1:0.01) to 100% mobile phase B (ethyl acetate) at a flow rate of 1 mL min^−1^. Each constituent’s retention time and ultraviolet/visible spectrum were compared with published authentic data to further confirm the peak identity [8]. At least three replicates were performed for each sample.

For liquid chromatography–tandem mass spectrometry (LC-MS/MS) analysis, the extracted carotenoid samples were dried and re-dissolved into 90% acetonitrile for HPLC separation (LC-30AD, Shimadzu, Kyoto, Japan) using a Ultisil AQ-C18 column (2.1 × 100 mm, 3 μm, Welch, West Haven, CT, USA). Mobile phases were 0.1% (*v*/*v*) formic acid in 100% ultrapure water (A) and 100% acetonitrile (B). The eluent flow was set at 0.4 mL min^−1^. The gradient started at 90% B and 10% A, changed to 100% B over 10 min, held for 2 min, and then returned to a mixture of 90% B and 10% A.

The MS/MS detection was performed on a 4600 TripleTOF mass spectrometer (Sciex, Framingham, MA, USA) using an APCI source, operated in positive ion mode. The detection range of primary mass spectrometry was 100–1500 *m*/*z*, and the scanning range of secondary mass spectrometry was 100–1250 *m*/*z* with a resolving power of 25,000. The sample injection volume was 1 µL. The ion source temperature was 550 °C with the atomizing and aux gas flow rates at 55 psi and the curtain gas flow rate at 35 psi.

### 2.5. Phylogenetic Analysis

Amino acid sequences of SyneCrtO, PaCrtZ, and the CrtZ from *Haematococcus pluvialis* (HpCrtZ, GenBank accession No. AKQ20654.1) were used as queries to search GenBank for their homologs in bacteria, red algae, heterokonts, fungi, and Viridiplantae using the BlastP algorithm with the cut-off criterion *E*-value < 0.05 [38,39]. For each search, when there were more than 250 hits, only the top 250 sequences were used in this study. A multisequence alignment was conducted with MUSCLE with default settings as implemented in MEGA 11 [40]. The phylogenetic tree was constructed using the IQ-Tree program (http://iqtree.cibiv.univie.ac.at/, accessed on 14 August 2023) with default settings (Substitution model: Auto; Bootstrap analysis: Ultrafast; Number of bootstrap alignments: 1000) and manually edited with MEGA 11 [40,41]. A Newick file, which can be opened with software such as MEGA 11, of the phylogenetic tree generated with all the sequences is provided as Supplemental File S1. A fasta file containing sequence information of all the homologs is provided as Supplemental File S2.

Sequence identity was calculated using the MegAlign program of the Lasergene package (DNASTAR, Madison, WI, USA).

### 2.6. Statistical Analysis

GraphPad Prism 7 (GraphPad Software, San Diego, CA, USA) was used for statistical analysis. To determine statistical significance, we employed Student’s *t*-test. Differences were considered significant at *p* < 0.05. Data are shown as the mean ± SE of at least three replications.

## 3. Results and Discussion

### 3.1. Synechocystis Expressing HpBKT Alone Did Not Synthesize Astaxanthin

*Synechocystis* is capable of synthesizing zeaxanthin in its cells, and its carotene β-hydroxylase SyneCrtR is the enzyme that catalyzes the conversion from β-carotene to zeaxanthin. To test whether HpBKT could utilize zeaxanthin for directly synthesizing astaxanthin, we first engineered *Synechocystis* to overexpress *HpBKT* driven by the promoter of the gene encoding Rubisco large subunit (*RbcL_pro_*). The *RbcL_pro_*:*HpBKT* transgene interrupted the endogenous *SyneCrtO* gene and introduced the kanamycin resistance as a selection marker (Figure 2A). The successful transgenic strains (namely *BKT*^+^/*δCrtO*) were picked from BG11 plates containing kanamycin, and further confirmed using a PCR (Figure 2B).

**Figure 3:**
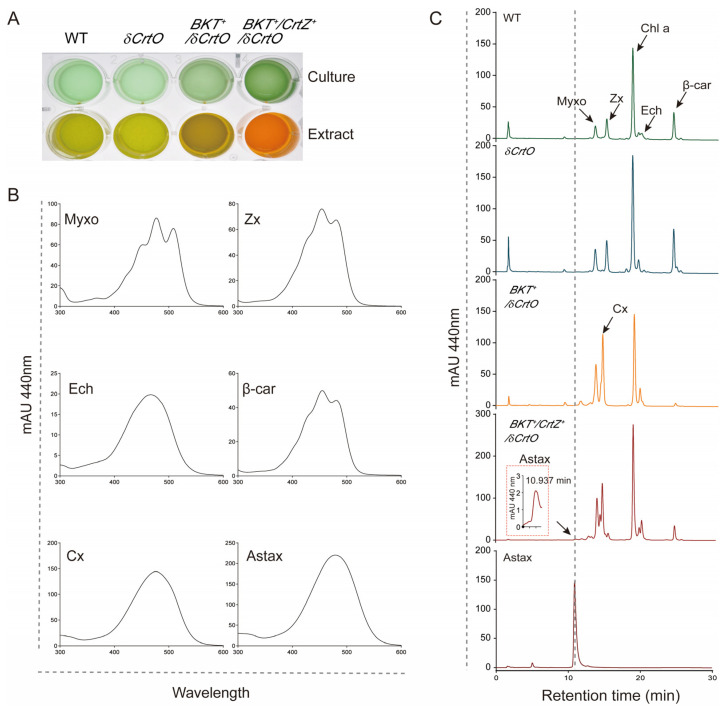
Pigment profile of different transgenic *Synechocystis* strains. (**A**) Representative cultures (upper panel) and pigment extracts (lower panel) from different strains growing at the logarithm stage (OD_750_ = 1.0). Cells and extracted pigment were placed in a 96-well plate for photography. (**B**) Absorption spectra of different carotenoid species (abbreviated as indicated in Figure 1) identified in this study. (**C**) HPLC separation profiles of total carotenoids extracted from different strains. Authentic astaxanthin was analyzed in parallel for comparison. An inset in the profile of the *BKT*^+^/*CrtZ*^+^/*δCrtO* strain shows the peak corresponding to astaxanthin.

The pigment analysis of the transgenic strains showed that the wild-type (WT) *Synechocystis* was able to synthesize β-carotene, zeaxanthin, myxoxanthophyll, and echinenone (Figure 3). No canthaxanthin, but only echinenone, was identified in the WT strain cells, indicating that SyneCrtO was only able to add the keto group to one of the unsubstituted β-rings of β-carotene (Figure 1 and Figure 3). This is different from the CrtO from *Haematococcus pluvialis*, which was reported to facilitate the biosynthesis of astaxanthin with two keto groups in cyanobacterium [42]. The *δCrtO* strain could not synthesize echinenone anymore (Figure 3). This was in line with our previous report that *SyneCrtO* could be silenced for simplifying the carotenoid metabolic pathway [27]. The silencing of *SyenCrtO* also left *SyneCrtR* as the only endogenous carotenoid oxygenase (Figure 1).

The *BKT*^+^/*δCrtO* cells growing in the BG11 medium demonstrated that the introduction of *HpBKT* resulted in an accumulation of canthaxanthin at the cost of both β-carotene and zeaxanthin (Figure 3). No astaxanthin was detectable in our study after several attempts with increased cultural volumes and altered growth conditions (Figure 3). Therefore, there might be two possibilities. First, HpBKT alone, or the combination of HpBKT and the endogenous SyneCrtR, was insufficient for the production of astaxanthin in *Synechocystis*. However, because of the existence of the newly synthesized canthaxanthin, it is obvious that HpBKT is capable of catalyzing the ketolation of β-carotene to produce canthaxanthin. This does not exclude the possibility that HpBKT could also use zeaxanthin as its substrate for astaxanthin production, but the detour of β-carotene for canthaxanthin resulted in a decreased level of zeaxanthin for its utilization. This raised the second possibility that HpBKT might possess a stronger preference for using β-carotene, compared with zeaxanthin, as its substrate. Such a preference is similar to the activity of the capsanthin/capsorubin synthase (CCS) in chili pepper and tiger lily (*Lilium lancifolium* Thunb. ‘Splendens’), which prefers antheraxanthin as its substrate for synthesizing capsanthin, but is also capable of using the further-oxygenated violaxanthin to synthesize capsorubin [12,43]. Moreover, our results also indicated that the endogenous carotene hydroxylase SyneCrtR could not use canthaxanthin as its substrate to synthesize astaxanthin, i.e., β-carotene is the specific substrate for SyneCrtR (Figure 3).

### 3.2. PaCrtZ Complemented the Biosynthesis of Astaxanthin in HpBKT-Transformed Cells

To confirm our postulation that SyneCrtR was not able to hydroxylate canthaxanthin, we co-expressed *HpBKT* with an additional *CrtZ*-type carotene 3,3′-hydroxylase gene from the bacterium *Pantoea agglomerans* (*PaCrtZ*), which was reported to use canthaxanthin as a substrate [44].

**Figure 4:**
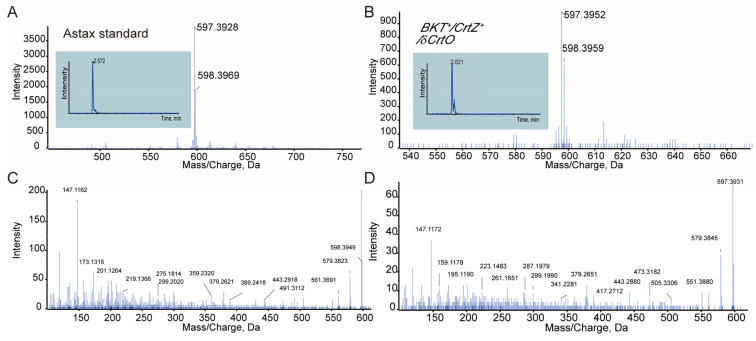
Identification of astaxanthin using LC-MS/MS. (**A**,**B**) Chromatograms (insets) and primary mass spectra of authentic astaxanthin (**A**) and the fraction collected from *BKT*^+^/*CrtZ*^+^/*δCrtO* strain extract (**B**). (**C**,**D**) Secondary mass spectra of the standard (**C**) and the collected fraction (**D**), respectively.

Similar to the *BKT*^+^/*δCrtO* strain, our transgenic *Synechocystis* strain overexpressing both *HpBKT* and *PaCrtZ* (namely *BKT*^+^/*CrtZ*^+^/*δCrtO*) was also confirmed using PCR amplification (Figure 2). Pigments were extracted from the mutant and analyzed using HPLC. Compared with authentic astaxanthin, a small peak sharing an identical retention time and absorbance spectrum was identified from the elution profile (Figure 3). We further collected the fraction and subjected the sample to LC-MS/MS detection. The results again confirmed that astaxanthin was obtained from the *BKT*^+^/*CrtZ*^+^/*δCrtO* strain that co-expressed *PaCrtZ* with *HpBKT* (Figure 4). These results also confirmed that PaCrtZ, but not the endogenous SyneCrtR, is able to catalyze the hydroxylation of canthaxanthin to generate astaxanthin.

### 3.3. Heterokont CrtZs Might Have Different Catalytic Properties

CrtR and CrtZ are two major classes of carotene hydroxylases identified in photosynthetic organisms, as well as in fungi and bacteria. Because we found that SyneCrtR was unable to catalyze the hydroxylation of canthaxanthin, we tried to determine the evolutionary divergence of these two classes of hydroxylases. From our sequence search and phylogenetic analysis (Supplemental Files S1, S2), it was clear that the homologs of PaCrtZ and HpCrtZ showed a close relationship by forming a mixed clade in the phylogenetic tree, whereas the homologs of SyneCrtR formed an isolated clade in the tree (Supplemental File S1). However, we found two CrtZ homologs of heterokonts, *Hondaea fermentalgiana* of Bigyra (HfCrtZ) and *Chaetoceros tenuissimus* of Ochrophyta (CtCrtZ), which stayed within the CrtR clade in the phylogenetic tree (Supplemental File S1). Sequence comparison showed that, although both HfCrtZ and CtCrtZ possess the highest sequence identities with the bacterial PaCrtZ, HfCrtZ also shares a higher identity with SyneCrtR compared with HpCrtZ and PaCrtZ (Table 1). Considering the complicated evolutionary scenarios, including horizontal gene transfer events, of heterokonts after the secondary endosymbiosis [45], it would be interesting to test whether HfCrtZ and CtCrtZ have different catalytic activities, which might help to better understand the evolution of heterokonts.

### 3.4. Synechocystis Growth Was Not Affected by Astaxanthin Biosynthesis

**Figure 5:**
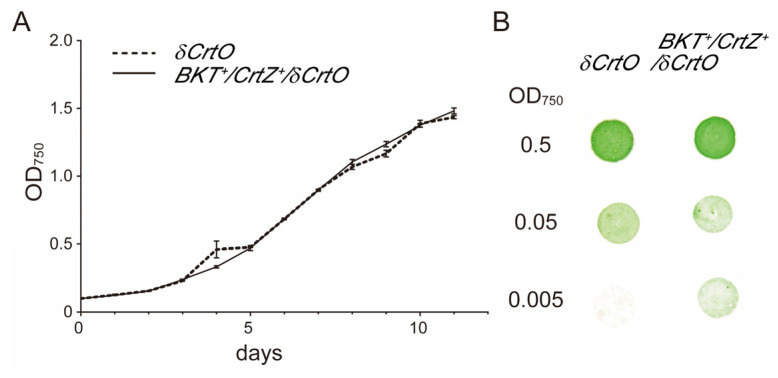
Growth of different transgenic *Synechocystis* strains. (**A**) Growth curves of the *δCrtO* and *BKT*^+^/*CrtZ*^+^/*δCrtO* strains during a 12 d course. Cells were cultivated in BG11 liquid medium with kanamycin at 50 μg mL^−1^ under normal conditions, and OD_750_ was measured daily. Error bars represent the standard error (*n* = 3). No significant difference (at *p* < 0.05 level, Student’s *t*-test) between the two strains was detected at each time point. (**B**) Growth of different strains on plates. Cells at the logarithm stage (OD_750_ = 0.5) were diluted in a 10 × series and spotted onto BG11 plates containing kanamycin at 20 μg mL^−1^. The photograph was taken after 10 d of growth in an incubator at normal growth conditions.

Cell growth is a key factor that might affect the heterologous production of natural products. In order to assess whether the modification of the carotenoid biosynthesis pathway for astaxanthin production had an impact on the growth of transgenic *Synechocystis* cells, we quantified the cell densities of the astaxanthin-producing *BKT*^+^/*CrtZ*^+^/*δCrtO* strain and the *δCrtO* (as a control) strain. Cells of both strains were cultured in BG11 liquid medium at 28 °C, and OD_750_ was determined. Our results indicated that these two strains had similar growth curves with no significant difference from the initiation of the cultivation to the logarithmic phase (Figure 5). Therefore, it is likely that the supplement of HpBKT and PaCrtZ to the endogenous carotenoid metabolic pathway, and the accumulation of canthaxanthin and astaxanthin, did not have a significant impact on the growth of *Synechocystis* cells under normal growth conditions.

## 4. Conclusions

In this study, we clarified the catalytic properties of SyneCrtO, SyneCrtR, and HpBKT for astaxanthin biosynthesis in the *Synechocystis* chassis. In summary, our results suggested that SyneCrtO is only able to introduce a keto group to one ring of β-carotene, that both SyneCrtR and HpBKT have the preference to use β-carotene as their substrate for hydroxylation and ketolation reactions to produce zeaxanthin and canthaxanthin, respectively, and that the endogenous CrtR-type carotene β-hydroxylase is not able to catalyze the 3,3′-hydroxylation of canthaxanthin to generate astaxanthin.

From our results, a higher accumulation of canthaxanthin and a much lower level of astaxanthin in *BKT*^+^/*CrtZ*^+^/*δCrtO* cells indicated that the bottleneck of astaxanthin biosynthesis exists at the canthaxanthin hydroxylation step. The novel conclusion indicated that a comprehensive screening or genetic engineering of CrtZs and other classes of carotene hydroxylases, except for CrtRs, for significantly enhanced catalytic activities should help to promote astaxanthin productivity in the cyanobacterial chassis. Considering the advantage of being able to grow both autotrophically and heterotrophically, the industrial production of astaxanthin in cyanobacteria through a synthetic biology approach could be a novel source of natural astaxanthin, replacing the cultivation of *Haematococcus pluvialis* as an antioxidant for the cosmetic and pharmaceutical markets.

## Figures and Tables

**Table 1:** Sequence distances among CrtR and CrtZs ^1^.

	SyneCrtR	PaCrtZ	HpCrtZ	HfCrtZ	CtCrtZ
SyneCrtR	100				
PaCrtZ	9.7	100			
HpCrtZ	12.4	38.4	100		
HfCrtZ	15.9	26	26.7	100	
CtCrtZ	12.4	19.5	25.6	34.6	100

^1^ Sequences are CrtR from *Synechocystis* (SyneCrtR) and CrtZs from the bacterium *Pantoea agglomerans* (PaCrtZ), the heterokonts *Hondaea fermentalgiana* (HfCrtZ) and *Chaetoceros tenuissimus* (CtCrtZ), and the green alga *Haematococcus pluvialis* (HpCrtZ). Sequence distance was calculated using the MegAlign program of the Lasergene package.

## Data Availability

Data are available on request from the authors.

## Supplementary Materials

The following supporting information can be downloaded at: https://www.mdpi.com/article/10.3390/antiox12101826/s1. Table S1. Primers used in this study. File S1. Phylogenetic tree in Newick format of all CrtR and CrtZ homolog sequences used in this study. File S2. Sequence information of all homolog sequences in Supplemental File S1 in fasta format.
