# Supplementary material for: Heterologous Production in the Synechocystis Chassis Suggests the Biosynthetic Pathway of Astaxanthin in Cyanobacteria
Source: Antioxidants (Basel). 2023 Oct 3;12(10):1826. doi: 10.3390/antiox12101826 (PMC10604110; doi:10.3390/antiox12101826)
Supplement: Supplementary file 1 [file antioxidants-12-01826-s001.zip › Supplemental Table S1.pdf]

Table S1. Primers used in this study.

Name	Sequence (5'-3')
F_{CrtZ}	atgctagtaaatagtttaatcgtcatcttg
R_{CrtZ}	ttattcgggcgaagacgacga
F_{CrtO}	gctattcaatgtttatggaggactgacctag
R_{CrtO}	accaatacccgtttgacggtttg
RbcLpro-F	tcaccatttggacaaaacatcagg
RbcLpro-R	ctaggtcagtcctccataaacattgaa
PsbA2pro-F	ggtatatggatcataattgtatgcccgact
PsbA2pro-R	ttggttataattccttatgtatttgtcgatgttcaga
